# Preventing pressure injuries in individuals with impaired mobility: Best practices and future directions

**DOI:** 10.26502/jsr.10020455

**Published:** 2025-07-08

**Authors:** Amber Peterson, Marcel P Fraix, Devendra K Agrawal

**Affiliations:** 1 Departments of Translational Research, College of Osteopathic Medicine of the Pacific, Western University of Health Sciences, Pomona, California 91766 USA; 2 Physical Medicine and Rehabilitation, College of Osteopathic Medicine of the Pacific, Western University of Health Sciences, Pomona, California 91766 USA

**Keywords:** Assistive technology, Impaired mobility, Incontinence-associated dermatitis, Nutritional support, Pressure injury, Pressure mapping, Preventive care, Rehabilitation medicine, Risk assessment, Skin integrity

## Abstract

Pressure injuries, also known as decubitus ulcers or bedsores, are a major source of preventable morbidity among individuals with impaired mobility, particularly those recovering from spinal cord injury, stroke, or traumatic brain injury. These wounds not only prolong hospital stays and increase healthcare costs but also significantly impair rehabilitation outcomes and quality of life. This paper provides a comprehensive review of the pathophysiology and risk factors underlying pressure injury development, emphasizing the unique vulnerabilities of patients with sensory loss, malnutrition, obesity, and incontinence. It synthesizes current best practices in prevention, including repositioning schedules, the use of pressure-redistributing support surfaces, moisture control, and nutritional optimization. The role of risk assessment tools such as the Braden Scale is examined alongside newer technologies like pressure mapping systems, Artificial intelligence-based prediction algorithms and biofeedback tools enhance individualization of care. Pharmacologic strategies and wound management principles, including debridement and antimicrobial use, are discussed in the context of multidisciplinary rehabilitation. Implementation challenges such as staffing constraints, variability across care settings, and financial limitations are addressed, and future directions are proposed to better integrate skin integrity metrics into functional outcome measures. Ultimately, this review advocates for a proactive, interdisciplinary approach that aligns preventive strategies with personalized care and emerging technology, positioning pressure injury prevention as a core component of high-quality, value-based medicine.

## Introduction

Affecting an estimated 1 in 3 million people in the United States annually, pressure injuries–also known as pressure injuries or decubitus ulcers–are a significant source of morbidity, particularly in patients with impaired mobility [1]. Incidence of pressure injuries vary by clinical setting, with the highest rates observed among patients in the orthopedic and critical care units [2]. Moreover, there is a direct correlation between the length of hospital stay and incidence of pressure injury, highlighting the importance of early and continuous prevention strategies during hospitalization and rehabilitation [3,4].

In the field of Physical Medicine and Rehabilitation (PM&R), populations at particularly high risk include individuals with spinal cord injury (SCI), stroke, and traumatic brain injury (TBI), who often experience reduced sensation, impaired mobility, and prolonged bedrest. Beyond the immediate physical burden, pressure injuries are associated with longer hospital stays, increased risk of infection, higher healthcare costs, and decreased quality of life [1,5,6]. From a rehabilitation perspective, they may also impede participation in therapy and delay functional recovery, making prevention a core responsibility of the interdisciplinary rehab team. Pressure injuries are not just localized skin injuries—they are systemic events with cascading impacts on rehabilitation potential, emotional well-being, and long-term outcomes.

Pressure injuries represent a serious and often preventable complication in patients with impaired mobility, with wide-ranging consequences that extend beyond just the skin. The economic burden is substantial: in the United States alone, the annual cost of managing pressure injuries is estimated to exceed **$26.8 billion**, accounting for both direct treatment costs and the extended length of hospital stays associated with complications such as infections, surgeries, and hospital readmissions [7]. For healthcare institutions, pressure injuries are considered a quality indicator, and hospital-acquired pressure injuries (HAPUs) are classified as “never events” by the Centers for Medicare and Medicaid Services (CMS), meaning that additional costs related to their treatment are often non-reimbursable [8].

From a clinical standpoint, pressure injuries are associated with **significant morbidity**, including localized pain, risk of cellulitis, osteomyelitis, and systemic sepsis [9,10]. Deep tissue ulcers may require surgical debridement, flap reconstruction, or long-term wound care, often complicating rehabilitation and delaying discharge [11]. For patients with SCI, stroke, or TBI, these ulcers can lead to **prolonged bedrest**, disruption of therapy schedules, and regression of functional gains.

The **quality-of-life impact** is equally profound. The presence of a pressure injury can significantly limit independence and community reintegration, particularly when assistive equipment such as wheelchairs or orthotics cannot be used due to wound location. In those with chronic wounds, the psychological burden—including anxiety, feelings of helplessness, and altered body image—can be comparable to that seen in other serious chronic illnesses [12].

This review aimed to examine the development of pressure injuries in individuals with impaired mobility, synthesize evidence-based prevention strategies, identify knowledge gaps, and emphasize the need for proactive, interdisciplinary prevention efforts throughout the rehabilitation continuum.

## 2. Pathophysiology of Pressure injuries

Pressure injuries often result from prolonged pressure, shear, or friction over bony prominences, resulting in ischemic damage of the skin and underlying tissues [13–15]. A common mechanism is sustained tissue pressure exceeding capillary filling pressure, leading to microcirculatory occlusion, ischemia, tissue death, and eventual ulceration [16,17]. The lower back and sacral regions are among the most frequent sites affected [18], particularly in individuals with impaired mobility who spend extended periods sitting or lying down. Research by Schubert et al. and Thorfinn et al. has shown that individuals with chronic SCI exhibit both elevated sitting pressures and delayed blood flow recovery in response to localized pressure, highlighting a physiologic vulnerability that places them at increased risk for developing pressure injuries [19,20].

**Figure 1:**
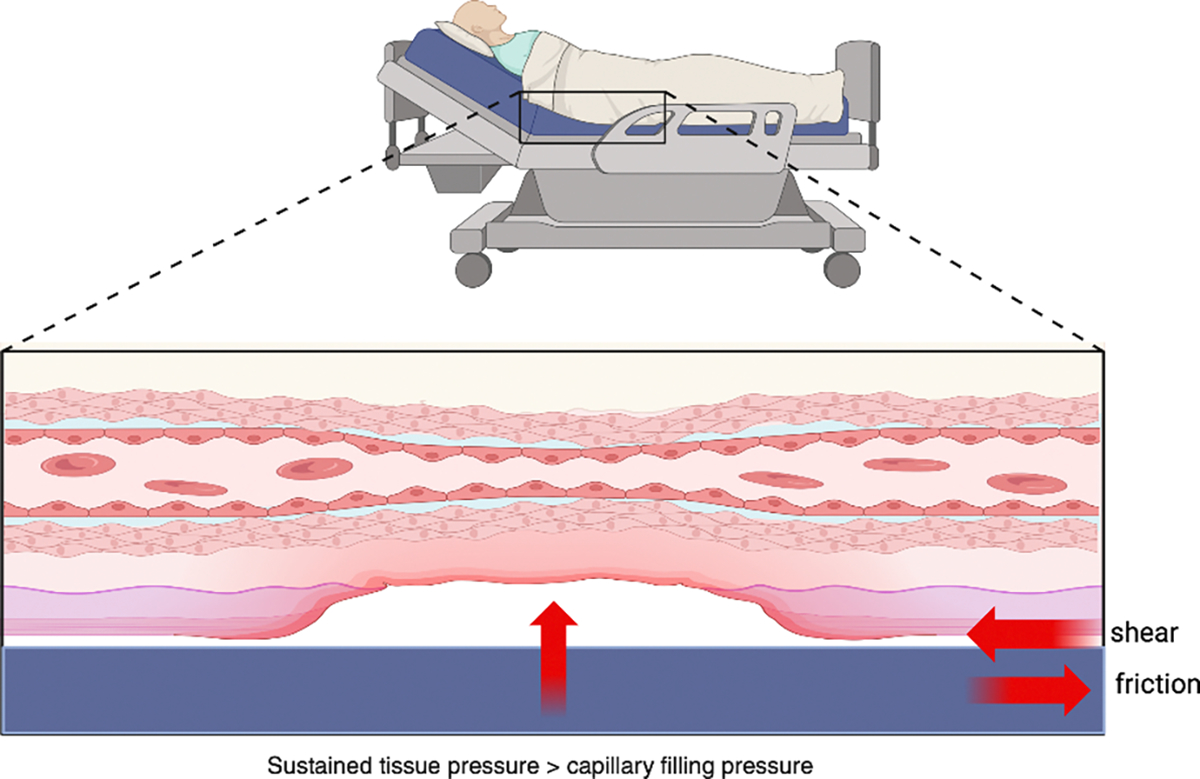
Mechanisms of Pressure Injury Formation: Sustained external pressure exceeding capillary filling pressure leads to microcirculatory occlusion, ischemia, and tissue necrosis. Shear and friction forces, such as those from immobility or poorly fitted devices, further compromise tissue perfusion and contribute to ulcer development.

Beyond sustained pressure, **shear, moisture, and friction** are key extrinsic factors that contribute to the pathogenesis of pressure injuries, often compounding the effects of ischemia (Figure 1). **Shear forces**—commonly encountered when the head of the bed is elevated or during transfers—cause distortion and angulation of the subdermal vasculature, leading to capillary occlusion and deep tissue damage even in the absence of overt skin breakdown [21–23]. **Friction**, typically occurring at the interface between the skin and external surfaces such as bed linens, wheelchairs, or orthotics, compromises the stratum corneum, increasing the risk of superficial skin erosion and facilitating the entry of microorganisms [24]. **Moisture**, particularly from urinary or fecal incontinence, perspiration, or wound exudate, macerates the skin and reduces its tensile strength, rendering it more susceptible to both friction and pressure-induced injury [25,26]. These biomechanical and environmental stressors often act synergistically—moist, fragile skin is more prone to friction, and shear can intensify pressure-induced ischemia—making comprehensive skin care and positioning strategies essential in at-risk populations.

## 3. Risk factors

**Figure 2:**
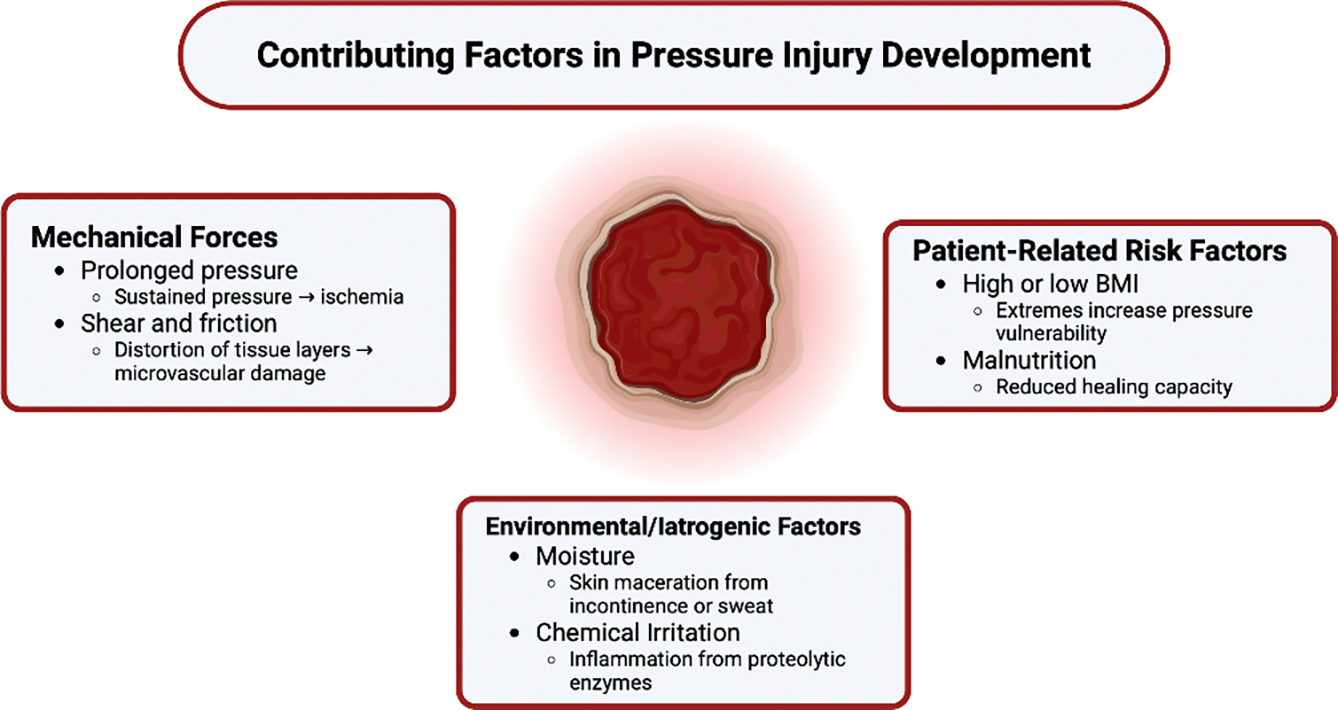
Contributing Factors in Pressure Injury Development. Multiple intrinsic and extrinsic factors contribute to pressure ulcer development, including mechanical forces, patient characteristics, and environmental exposures that impair tissue integrity and perfusion.

Several patient-specific and clinical conditions significantly increase susceptibility to pressure injury, notably **immobility**, **body mass-index (BMI)**, and **nutrition** (Figure 2). **Prolonged surgical procedures**, particularly those lasting more than three hours, are associated with a markedly increased risk of pressure injury due to sustained tissue ischemia under general anesthesia [27]. During surgery, patients are often immobile, hemodynamically vulnerable, and exposed to unrelieved pressure over bony prominences such as the sacrum, occiput, or heels. Furthermore, muscle relaxation and anesthesia reduce the normal protective responses of the body to offload pressure [28,29].

Sensory loss is a critical yet often underappreciated factor in pressure injury formation. Individuals with intact sensation and mobility can consciously or unconsciously shift positions to offload points of increased pressure. Baliki and Apkarian [30] propose that **nociception operates continuously, even in the absence of pain perception, serving as a subconscious protective system** that guides behavior to prevent tissue damage. This form of nociception influences habitual actions like shifting posture or adjusting gait without rising to conscious awareness. These behaviors are shaped by prior learning and reinforced through subtle nociceptive input over time. In individuals with SCI, this protective mechanism is disrupted due to the loss of sensory input below the level of injury. As a result, patients may fail to offload pressure points instinctively, placing them at heightened risk for pressure injuries. Baliki and Apkarian [30] highlight how the **absence of nociceptive feedback**, rather than the absence of pain alone, can lead to self-destructive behaviors—paralleling what is observed in other conditions with impaired pain perception, such as leprosy or painless channelopathies. These insights suggest that pressure injury formation in patients with sensory loss may, in part, stem from a **loss of unconscious nociceptive-driven behaviors that normally protect against sustained pressure and tissue damage**. This constant load exceeds capillary perfusion pressure, compromising blood flow and oxygen delivery to tissues. Without spontaneous repositioning or assisted turning, even short periods of sustained pressure can result in irreversible damage to the dermis and underlying muscle [31].

**Obesity** is an independent risk factor for pressure injury development [32,33]. Excess body mass increases interface pressures at weight-bearing sites, while the abundance of soft tissue can impair capillary flow, heightening the risk of deep tissue injury [34,35]. Additionally, adipose tissue tends to be poorly oxygenated, which delays tissue recovery following ischemic episodes [36,37]. Obesity is also closely linked to higher rates of diabetes, further compounding the risk of pressure wound formation and impaired healing [38,39]. These factors, combined with the increased difficulty of repositioning and reduced mobility often seen in obese patients, make pressure injury prevention particularly challenging in this population.

Interestingly, a study by Chen et al found a U-shaped relationship between BMI and pressure injury risk, with the lowest risk observed among overweight patients (BMI 27.5 kg/m^2^), and the highest risk among both underweight and obese individuals [40]. This pattern demonstrates the complex relationship between body composition and skin integrity, suggesting that both insufficient and excessive body mass can interfere with the ability of the skin to tolerate sustained pressure. The authors note that the greatest incidence of pressure injuries occurred in underweight patients, likely reflecting the effects of poor nutritional reserves and the diminished ability of the body to withstand catabolic stress. In these individuals, there is often inadequate subcutaneous padding, less protein reserve, and limited regenerative capacity, all of which contribute to higher vulnerability.

**Malnutrition** itself significantly impairs the body’s ability to maintain and repair tissue [41,42]. It is particularly prevalent among the elderly, individuals with dysphagia following stroke, and patients with prolonged hospitalization––groups who are also at elevated risk for pressure injuries [43,44]. When nutritional intake is insufficient, the body lacks the raw materials needed for tissue regeneration, immune function, and the complex cellular signaling involved in wound healing. Protein-energy malnutrition disrupts key processes in wound healing by reducing collagen synthesis, angiogenesis, and fibroblast activity [45,46]. **Additionally,** deficiencies in key micronutrients such as **vitamin C**, **zinc**, and **prealbumin** further compromise the regenerative capacity of the skin and impair immune defense, compounding the challenge of healing in nutritionally at-risk patients [47,48].

**Beyond impaired nutrition, external factors such as moisture and chemical irritation also play a critical role in skin breakdown. Both** urinary and fecal incontinence introduce excess moisture and irritants to the skin. Enzymes such as proteases and lipases, found in stool and urine, compromise skin integrity by breaking down the stratum corneum [49]. Incontinence promotes **maceration of the skin**, reducing skin tensile strength and making it more susceptible to damage from shear and friction forces. Incontinence-associated dermatitis (IAD) is a distinct clinical entity characterized by inflammation and erosion of the skin due to prolonged exposure to urine or feces, and if not promptly addressed, it can serve as a precursor to full-thickness pressure injuries [50,51]. A histopathologic study in a rat model demonstrated that macerated skin exposed to proteolytic enzymes and bacteria exhibited inner tissue damage, with enzymatic invasion extending beyond the superficial layers—supporting the idea that incontinence leads not only to surface irritation but also to deeper tissue injury [52]. This is especially concerning in patients with neurogenic bladder or bowel dysfunction, such as those with SCI or TBI, where the combination of incontinence and immobility significantly heightens the risk of skin breakdown.

Although physiological stressors such as malnutrition and moisture compromise the skin’s structural resilience, successful prevention often hinges on the patient’s active participation by recognizing discomfort, requesting assistance, and adhering to repositioning or hygiene routines. When cognition is impaired, these self-protective behaviors quickly erode, exposing yet another vulnerable subgroup to unchecked pressure, shear, and chemical irritation. Patients with cognitive impairment face unique challenges in pressure injury prevention. Conditions such as **TBI**, **dementia**, **encephalopathy**, or **intellectual disability** impair a patient’s ability to perceive risk, communicate discomfort, follow instructions, and participate in routine care activities. In the acute setting, individuals with TBI may exhibit **disinhibition**, **impulsivity**, or **agitation**, interfering with regular repositioning or use of support surfaces [53]. In long-term care, patients with Alzheimer’s disease or other dementias may not recognize the need to change position or report early signs of skin breakdown. These patients may also resist care, pull off dressings, or be unaware of soiled garments, further increasing their vulnerability [54]. Cognitive impairment is often accompanied by **functional dependence**, **incontinence**, **malnutrition**, and **limited mobility**, creating a perfect storm for skin breakdown, and highlighting the need for interdisciplinary care.

### Staging of pressure injuries

The National Pressure Injury Advisory Panel (NPIAP) released a comprehensive revision in 2016 to the pressure injury staging definitions, encompassing stages 1 through 4, along with classifications such as unstageable pressure in juries and deep tissue pressure injuries [55].

- Stage 1 Pressure Injury: Non-blanchable erythema of intact skin
- Stage 2 Pressure Injury: Partial-thickness skin loss with exposed dermis
- Stage 3 Pressure Injury: Full-thickness skin loss
- Stage 4 Pressure Injury: Full-thickness skin and tissue loss
- Unstageable: Obscured full-thickness skin and tissue loss
- Deep Tissue Pressure Injury: persistent non-blanchable deep red, maroon or purple discoloration

These staging definitions play a critical role in standardizing the assessment and documentation of pressure injuries across healthcare settings. By clearly delineating stages based on the depth of tissue damage and visible characteristics, these definitions support accurate diagnosis, inform evidence-based treatment plans, and enable consistent communication among care teams. Additionally, proper staging is essential for tracking outcomes, meeting regulatory requirements, and determining reimbursement, as pressure injuries are often indicators of care quality.

### Assessment and Monitoring

Effective prevention and early intervention for pressure injuries rely heavily on systematic risk assessment and continuous monitoring. In immobile patients, who are often unable to recognize or report early tissue damage, objective tools and frequent evaluations are critical components of care. An effective assessment strategy integrates structured risk tools, routine skin evaluations, pressure-distribution technology, and thorough documentation embedded in quality assurance systems.

### 5.1. Risk Assessment Tools

The **Braden Scale for Predicting Pressure Sore Risk** is the most widely adopted risk assessment tool in hospitals and rehabilitation centers. It evaluates six domains—sensory perception, moisture, activity, mobility, nutrition, and friction/shear—each scored from 1 (severe impairment) to 4 (no impairment), with lower cumulative scores indicating higher risk [56]. A score ≤18 is commonly used as the threshold for initiating pressure injury prevention protocols. The Braden Scale has been validated across diverse patient populations and clinical settings, though its predictive accuracy may be reduced in specific populations such as those over the 60 years of age [57].

Other tools such as the **Norton Scale** and **Waterlow Score** are also used in some clinical settings, although they may offer varying reliability and specificity. In a prospective observational study of 250 surgical patients, Gurkan et al. compared the predictive performance of these three scales [58]. The Braden and Waterlow scales both demonstrated 100% sensitivity, effectively identifying all patients who went on to develop pressure injuries. However, their specificities were low (40.4% for Braden and 48.1% for Waterlow) indicating a high false positive rate. The Waterlow scale showed the highest overall accuracy, though the study also noted wide confidence intervals around relative risk estimates, limiting definitive conclusions. Given these limitations, some institutions supplement standard tools with population-specific models, such as SCI-specific risk models that account for sensory deficits, autonomic dysfunction, and reduced mobility, or ICU-adapted risk tools that incorporate factors like sedation [59,60]. Regardless of the tool selected, regular re-assessment remains critical, as risk profiles can shift rapidly due to changes in mobility, nutritional status, or acute illness.

### 5.2. Skin Inspection and Pressure Mapping

Routine skin checks are a frontline method for detecting early signs of pressure-related skin damage. These assessments should be performed on admission, with every shift, and after significant clinical changes. Particular attention should be paid to high-risk sites including the sacrum, heels, ischial tuberosities, greater trochanters, scapulae, and occiput. Early indicators of impending tissue damage may include non-blanchable erythema, localized warmth or coolness, induration, or skin texture changes, even in the absence of an open wound. Shi et al. demonstrated that non-blanchable erythema independently predicts development of deepening pressure injuries within 28 days [61]. In individuals with darker skin tones, discoloration may be subtler and require heightened vigilance.

Pressure mapping systems provide an objective complement to manual skin assessments. Utilizing sensor embedded cushions or mats, they generate real-time visualizations of interface pressure distribution, particularly valuable for wheelchair users or bed-bound patients. In a pilot study with SCI participants, *real-time pressure map feedback significantly improved users’ confidence* in performing effective weight shifts to offload pressure at high-risk zones [62]. A separate investigation of six wheelchair users utilizing a mobile app linked to a pressure mat demonstrated that pressure-map visualization helped clinicians review and tailor repositioning behaviors—highlighting hotspot detection—even when reminder adherence was modest [63]. In the ICU setting, **continuous bedside pressure mapping (CBPM)** equipped clinicians to visually identify and relieve high-pressure points, significantly reducing hospital-acquired pressure injury incidence (0.9% vs. 4.8%) [64]. Thus, when combined with clinical evaluation, pressure mapping enhances preventive precision—supporting both behavioral engagement and data-driven hotspot identification in real time.

Manual checks catch early, subjective signs like color and temperature changes, while mapping confirms pressure exposure and guides adjustment of seating or bedding. This multimodal approach ensures timely intervention and more effective pressure injury prevention.

### 5.3. Documentation and Quality Metrics

Equally important to prevention, high-quality documentation is vital not only for clinical continuity but also for meeting regulatory and quality improvement standards. At the bedside, this includes regular entries of Braden scores, skin inspection findings, repositioning frequency, support surface use, and wound staging. From an institutional perspective, pressure injury incidence and prevalence are key quality indicators that affect hospital reputation and reimbursement. The Centers for Medicare & Medicaid Services (CMS) classifies Stage 3, Stage 4, and unstageable hospital-acquired pressure injuries as “never events”—preventable occurrences that trigger payment penalties under the Hospital-Acquired Condition Reduction Program (HACRP) [8]. This policy emphasizes the importance of preventing pressure injuries, as their occurrence not only impacts patient health but also has significant financial implications for healthcare facilities.

To address this, hospitals and rehabilitation facilities have attempted to implement multidisciplinary skin integrity programs and integrated skin check flowsheets into electronic medical records [65]. Use of standardized staging terminology (per the NPIAP) and root cause analysis following new ulcer formation are now routine in many institutions [55,66]. Importantly, continuous education of nursing staff, caregivers, and patients is a cornerstone of sustainable improvement in pressure injury prevention and documentation.

## 6. Evidence-Based Prevention Strategies

**Figure 3:**
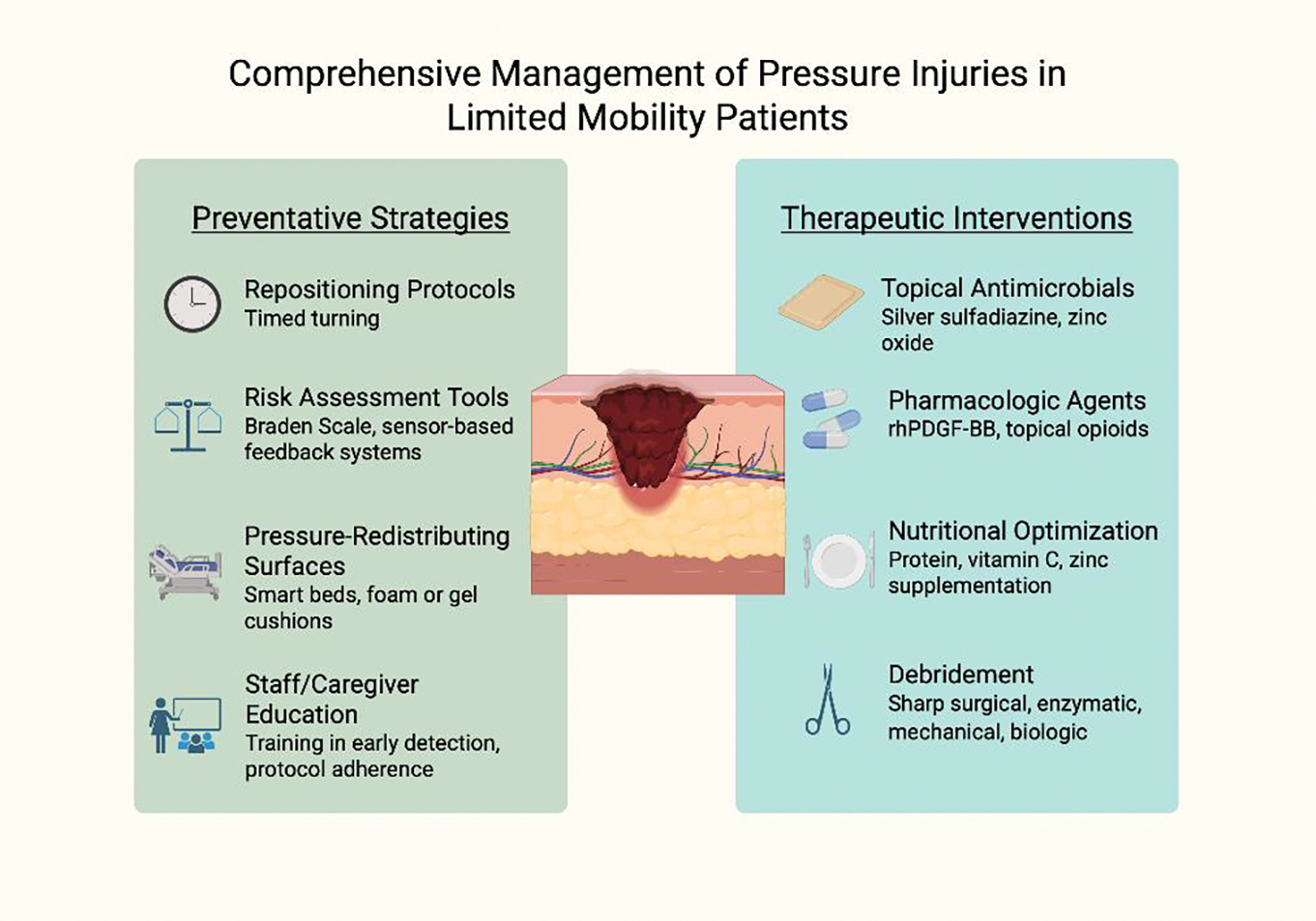
Comprehensive Management of Pressure Injuries in limited Mobility Patients. Effective pressure injury management involves both prevention and treatment, targeting biomechanical stressors, wound environment, systemic health, and caregiver competency to promote healing and prevent recurrence.

Best practices for pressure injury prevention and management emphasize a multidisciplinary approach that integrates risk assessment, early intervention, and consistent care protocols (Figure 3). Repositioning schedules, typically every two hours, are essential to redistribute pressure and minimize prolonged ischemia over bony prominences. The use of pressure-redistributing surfaces—including specialized mattresses and wheelchair cushions—further reduces tissue stress. Skin care protocols should prioritize moisture control, particularly in patients with incontinence, through regular cleansing and application of barrier products. Nutritional support also plays a vital role, as protein-energy malnutrition impairs wound healing and increases susceptibility to skin breakdown. Clinical implementation requires integration of these practices into daily workflows, supported by staff education, standardized documentation, and continuous quality monitoring. Institutions that adopt structured skin integrity programs, including regular skin assessments and root cause analysis after new ulcer development, have demonstrated reductions in pressure injury incidence and improved patient outcomes.

### 6.1. Repositioning Protocols and Scheduling

Repositioning is a fundamental, evidence-based intervention in the prevention of pressure injuries in patients with impaired mobility. The principle behind regular repositioning is to alleviate prolonged pressure over bony prominences—such as the sacrum, heels, and greater trochanters—that can exceed capillary closing pressure (~32 mmHg) and lead to tissue ischemia, cell death, and ultimately ulcer formation [67]. The commonly used protocol of repositioning every two hours (q2h) has been supported historically by clinical experience and expert consensus. According to the NPIAP, scheduled turning should be implemented as part of a comprehensive prevention plan, particularly in bed-bound patients [68].

Recent randomized controlled trials and observational studies have begun to challenge the universal application of the q2h rule. For example, the TURN study by Bergstrom et al. investigated turning intervals of 2, 3, and 4 hours in nursing home residents on high-density foam mattresses and found no significant difference in pressure injury incidence across groups [69]. This suggests that the efficacy of repositioning may depend on the support surface used and patient-specific factors, such as perfusion status, nutritional health, and comorbidities. Moreover, newer technologies like continuous bedside pressure mapping and wearable sensors are being explored to guide individualized turning schedules based on real-time risk profiling [70].

In rehabilitation settings, repositioning is a foundational component of therapeutic mobility planning for individuals with SCI, who often experience both immobility and impaired sensation. Because individuals with SCI often lack sensation in pressure-prone areas, scheduled pressure relief maneuvers—rather than relying on discomfort to prompt movement—are critical for preventing deep tissue injury. To prevent pressure injuries, clinical guidelines including from the Paralyzed Veterans of America recommend that wheelchair users perform scheduled pressure relief maneuvers, such as forward leans, lateral shifts, or wheelchair push-ups, every 15 to 30 minutes [71,72]. This frequency is based on earlier evidence showing that intermittent weight shifts replenish tissue oxygenation and reduce inflammatory markers associated with prolonged pressure. However, a study by Makhsous et al. comparing dynamic seating systems and wheelchair push-ups found that although push-ups effectively reduce interface pressure, they may not fully restore tissue perfusion, suggesting the need for more varied or sustained pressure relief strategies to optimize tissue health [71]. The study also highlights a key challenge: these repositioning maneuvers must be self-initiated, yet reliance on self-reported frequency of pressure reliefs is unreliable, as users tend to overestimate how often they perform them, emphasizing the need for objective monitoring and continued patient education.

While repositioning remains the gold standard for preventing pressure injuries, clinical guidelines emphasize the importance of incorporating patient-specific factors such as individual risk, skin condition, and support surface type when planning pressure relief schedules. Rather than relying solely on fixed time intervals, these considerations help tailor care within established repositioning protocols. Physical and occupational therapists are essential in this process, collaborating with patients to integrate these customized repositioning strategies into daily routines and fostering the development of habits that promote long-term independence and ulcer prevention.

### 6.2. Skin Care: Moisture Control and Barriers

Effective skin care is a cornerstone in pressure injury prevention, particularly in patients with limited mobility who are vulnerable to prolonged moisture exposure due to incontinence, sweating, or wound exudate. Moisture-associated skin damage (MASD) compromises the epidermal barrier, increasing the risk of maceration, friction, and shear—mechanisms known to accelerate pressure injury development [73].

Effective moisture management is a critical component in preventing pressure injuries, especially in immobile patients whose skin is frequently exposed to urine and feces. This prolonged exposure disrupts the skin barrier by increasing maceration, friction, and pH imbalance, predisposing patients to incontinence-associated dermatitis (IAD) and, ultimately, pressure injuries. Topical skin barriers are essential in protecting the skin from these harmful effects. Commonly used products include petrolatum-based ointments, zinc oxide pastes, and dimethicone creams. Zinc oxide forms a durable physical barrier and offers mild antiseptic properties, making it especially effective at enhancing wound healing in high-moisture areas such as the sacrum, perineum, and inner thighs [74]. For patients with established IAD, a retrospective study found that treatment with a combination of topical antibiotic (Biomycin) and antifungal (clotrimazole) resulted in complete healing in 61% of cases and significant improvement in an additional 22% [75], supporting the value of antimicrobial-based protocols in managing secondary skin infections. A pilot study further demonstrated that a stepwise approach using zinc oxide followed by petroleum jelly not only reduced IAD severity in older adults, but also improved skin hydration and normalized pH, both essential for maintaining skin integrity [76]. Similarly, Glass et al. found that incorporating specialized skin cleansers to standard IAD care enhanced healing within one week, particularly among patients with pre-existing skin damage [77]. These studies show the importance of a multifaceted skin care regimen that includes protective barriers, antimicrobial agents, and gentle cleansing to effectively manage and prevent IAD.

### 6.3. Pharmacologic Management

Pharmacologic strategies for pressure injury management are adjunctive to mechanical and supportive interventions but may play a critical role at different stages, from prevention to treatment of established wounds. These interventions target the underlying pathophysiology: impaired perfusion, inflammation, microbial invasion, and impaired cellular healing.

#### 6.3.1. Prevention: Limited but Evolving Role

Currently, there are no FDA-approved pharmacologic agents specifically indicated for the prevention of pressure injuries. However, emerging therapies explore potential adjunctive benefits, particularly in high-risk populations. Micronutrients such as zinc, vitamin C, and arginine have been used to promote skin integrity and mitigate oxidative stress, especially in individuals with poor nutritional status [78,79]. While evidence supporting their role in primary prevention remains limited, these agents may function as valuable supportive measures. Their potential to complement standard preventive strategies such as repositioning, moisture management, and pressure redistribution demonstrates a growing interest in multimodal approaches to pressure injury prevention.

#### 6.3.2. Early Ulcer Development: Modulating Inflammation and Bacterial Load

When pressure injuries are in early stages (Stages 1–2), timely intervention targeting inflammation and bacterial colonization is essential to prevent progression. Topical antimicrobials such as silver sulfadiazine, cadexomer iodine, and polyhexamethylene biguanide (PHMB) have demonstrated efficacy in reducing surface bioburden without impairing healing [80,81]. Particularly, silver-based dressings offer broad-spectrum antimicrobial coverage and are commonly used in ulcers at risk of infection or in immunocompromised individuals [82].

In addition to microbial control, modulating inflammation in the early phases is critical. A study by Peña et al. showed that early pressure injuries are often marked by subclinical inflammation and elevated cytokines such as TNF-α and IL-1β, which contribute to tissue damage before overt skin breakdown [83]. This was further supported by recent transcriptomic and proteomic profiling of patients with Stage II–IV pressure injuries, which revealed that greater ulcer severity correlated with systemic upregulation of inflammatory pathways, including TNF and IL-6 signaling [84]. These findings suggest that systemic immune dysregulation plays a role in pressure injury progression and that early anti-inflammatory intervention may help preserve skin integrity and prevent deep tissue damage.

#### 6.3.3. Established Ulcers: Promoting Healing and Controlling Infection

For Stage 3 or Stage 4 ulcers with clear signs of infection, systemic antibiotics may be necessary, particularly when there is cellulitis, osteomyelitis, or systemic signs of infection. Common agents include amoxicillin-clavulanate or clindamycin, and in cases involving MRSA or resistant organisms, doxycycline, trimethoprim-sulfamethoxazole, or linezolid may be used [85]. However, prophylactic antibiotics without evidence of infection are not recommended and may promote resistance, as studies have shown antibiotic-resistant and multi-drug-resistant bacteria isolated from pressure injuries [86–88].

Advanced ulcers may benefit from adjunctive pharmacologic agents that modulate wound healing. Recombinant human platelet-derived growth factor (rhPDGF-BB), a bioengineered glycoprotein that promotes angiogenesis and granulation tissue formation, is FDA-approved for diabetic foot ulcers and has been shown to be effective in pressure injury management [89–91]. In one randomized controlled trial involving full-thickness pressure injuries, patients who received rhPDGF-BB followed by salvage surgical repair had significantly improved one-year healing rates compared to those receiving placebo and surgery [92], suggesting that rhPDGF-BB may optimize the wound bed for surgical closure. Although most data supporting rhPDGF-BB come from studies on diabetic foot ulcers, where it has been shown to accelerate healing and increase the rate of complete closure [89,90], these findings may have translational relevance for similarly impaired healing in pressure injuries. However, although rhPDGF-BB demonstrates potential efficacy, its broader clinical application is constrained by a limited number of controlled studies in pressure injuries and the associated high cost [91].

Analgesics are essential in managing pain, particularly during debridement or dressing changes. A multimodal pain regimen—incorporating acetaminophen, NSAIDs, and topical analgesics—should be tailored to the individual patient’s needs to optimize pain control while minimizing side effects. For neuropathic or chronic pain, gabapentinoids or low-dose opioids may be appropriate, though these require cautious use, especially in older adults or patients with spinal cord injuries due to increased risk of adverse effects and dependency. Topical opioids have emerged as a promising option for painful pressure injuries, particularly in palliative care patients who wish to avoid systemic opioid therapy. Evidence suggests that topical morphine can significantly reduce ulcer-related pain and may decrease the need for systemic opioids, thereby improving quality of life in select patients [93].

### 6.4. Nutritional Optimization

Nutritional status is a critical determinant of skin integrity, immune function, and wound healing. Malnourished patients are significantly more likely to develop pressure injuries, particularly those with protein-calorie malnutrition or micronutrient deficiencies. The 2019 International Pressure Injury guidelines recommend a comprehensive nutritional assessment for at-risk patients, including measurement of weight trends, serum albumin/prealbumin levels, and dietary intake [94].

Adequate protein intake is essential to support collagen synthesis and immune function, which are vital for effective wound healing. While the Recommended Dietary Allowance (RDA) for adults is 0.8 g/kg/day of protein, older adults with reduced mobility often have increased needs due to changes in body composition and decreased physical activity. Studies suggest that a minimum of 1.0 g/kg/day of protein is necessary for elderly patients, with intake recommendations adjusted according to pressure injury severity [95]. Patients with Stage I and II pressure injuries generally benefit from 1.0–1.4 g/kg/day of protein, whereas those with more advanced Stage III and IV wounds may require 1.5–2.0 g/kg/day, with some guidance advocating up to 2.2 g/kg/day [96]. However, it is important to balance protein intake carefully in this vulnerable population to prevent placing undue stress on kidney function.

In addition to macronutrients, supplementation with micronutrients such as arginine, zinc, and vitamin C has been investigated for their potential to enhance pressure injury healing [78]. Although the evidence remains inconclusive, some studies suggest that these supplements may accelerate wound resolution [97]. Due to the complex and individualized nutritional needs of patients with reduced mobility, involvement of a registered dietitian is vital in both inpatient and outpatient rehabilitation settings. Dietitians play a crucial role in developing personalized nutritional plans that address metabolic demands and functional limitations, ultimately supporting optimal healing and prevention of further pressure injuries.

### 6.5. Debridement

Debridement plays a critical role in the management of pressure injuries in patients with limited mobility. This process, which may be enzymatic, mechanical, biological, or sharp surgical, involves the targeted removal of necrotic tissue, slough, and biofilm which can harbor bacteria and delay wound healing [98]. By converting a chronic wound into an acute one, debridement promotes new tissue formation and improves the effectiveness of topical treatments and systemic antibiotics [99,100].

Sharp surgical debridement offers a rapid and definitive method to clear large areas of devitalized tissue, especially when other methods fail or when there is a need to urgently reduce bacterial load to prevent local or systemic infection [101]. Timely surgical intervention can be lifesaving by mitigating complications such as cellulitis, osteomyelitis, or sepsis, all of which can derail a patient’s rehabilitation trajectory and prolong immobility [102]. Early and appropriate debridement can also facilitate accurate wound staging and assessment, allowing for more tailored and effective treatment planning. In the context of rehabilitation, this can be critical not only for wound healing but also for maximizing mobility, independence, and quality of life.

### 6.6. Staff Education and Patient/Caregiver Training

Education is foundational in implementing effective pressure injury prevention strategies. Multiple studies have demonstrated that structured education programs for nurses, aides, therapists, and family caregivers result in improved knowledge, higher compliance with prevention protocols, and reduced incidence of pressure injuries [103–105]. A randomized controlled trial by Uzun et al. showed that individualized, face-to-face training significantly increased caregivers’ perineal care knowledge compared to brochure-based education, suggesting that direct instruction with caretakers may lead to more effective and consistent moisture management [103]. Educational interventions should include training on proper repositioning techniques, use of pressure-relieving equipment, early signs of skin breakdown, and moisture management. In rehabilitation settings, education must be interdisciplinary and sustained, as patients are often preparing for long-term home care or assisted living transitions. Engaging patients and caregivers in shared decision-making and skill-building such as teaching pressure relief maneuvers for wheelchair users or how to inspect skin daily empowers self-management and promotes long-term skin integrity.

## 7. Technology and Innovation

As pressure injury prevention and management evolve, the integration of novel technologies into clinical practice—particularly within rehabilitation medicine—has shown promising potential to enhance patient monitoring, individualized risk assessment, and therapeutic interventions. These innovations are particularly beneficial in PM&R settings where patients often have long inpatient stays and complex mobility limitations.

### 7.1. Pressure-Relieving Surfaces

The selection of appropriate pressure-relieving surfaces is critical in preventing pressure injuries in immobile patients. These surfaces aim to redistribute pressure away from bony prominences and reduce the duration and intensity of pressure exposure. High-specification foam mattresses and alternating pressure air mattresses have demonstrated efficacy in reducing pressure injury incidence compared to standard hospital mattresses [106]. Similarly, for seated patients, specialized wheelchair cushions made from air, gel, foam, or hybrid materials help reduce pressure and shear forces [107,108].

Research has shown that inappropriate seating systems lead to increased interface pressure over vulnerable areas such as the ischial tuberosities and sacrum, elevating the risk of pressure injuries [108]. Foam cushions, particularly contoured high-density foams, offer postural stability and are effective when appropriately matched to the user’s anatomy. Hybrid cushions combine materials to optimize both pressure relief and postural control. Importantly, clinical studies highlight that no single cushion type is ideal for every patient. A randomized crossover study by Burns and Betz found that interface pressures varied significantly depending on cushion type [109], reinforcing the need for individualized assessment. Surface selection should be individualized, considering factors such as patient weight, mobility, moisture risk, and personal preference, and should be re-evaluated regularly as patient status changes. Additionally, as numerous studies point out in their assessment of wheelchair cushions, a properly fitting wheelchair is as important, if not more important, as the cushion itself. Improper seat dimensions or back support can create abnormal pressure points, promote sacral sitting, and increase sheer, all which can contribute to tissue breakdown [110].

The importance of a properly fitting assistive device extends beyond wheelchair use, as evidenced by the high prevalence of stump ulcers among amputees. These ulcers can significantly impair mobility and quality of life. Amputees face a distinct challenge, as the soft tissue of the residual limb lacks the durability of the plantar surface and is less capable of withstanding comparable weight-bearing forces [111]. Therefore, prosthetic liners are used to provide additional support and cushioning to the stump. The role of prosthetic liner design and material properties in either contributing to or mitigating ulcer formation remains poorly defined. As Klute et al. highlight, while liner material properties have been extensively studied in laboratory settings, their actual influence on functional outcomes and in vivo performance has yet to be clearly established [112]. This gap in evidence shows the need for research that links liner selection to patient-centered outcomes such as ulcer prevention, comfort, and long-term skin integrity.

### 7.2. Smart Beds and Wearable Sensors

Smart surfaces, such as pressure-redistributing beds equipped with real-time pressure mapping, represent a significant advancement in the prevention of pressure injuries in immobile patients. These systems use embedded sensors to monitor pressure distribution continuously and can prompt nursing staff when repositioning is needed, reducing reliance on fixed turn schedules. Some advanced systems even incorporate automatic offloading or subtle, autonomous position changes to relieve pressure without disrupting the patient’s rest. In parallel, wearable sensors placed at high-risk bony prominences track pressure duration and skin microclimate (temperature, humidity), providing clinicians with early alerts before tissue damage occurs [113]. These technologies have shown promise in improving adherence to offloading protocols and enabling earlier intervention.

However, despite their potential, current evidence highlights uncertainty regarding the relative effectiveness of different support surface types. A 2021 Cochrane review of 41 randomized controlled trials found that while some high-specification foam mattresses reduced pressure ulcer incidence compared to standard foam, the comparative benefits of alternating pressure (active) air surfaces versus reactive surfaces (such as fiber, gel, or water-based) remain unclear [106]. Active air surfaces may reduce ulcer risk in certain settings, such as the operating room, but may not outperform reactive air surfaces in nursing homes [114,115]. Moreover, although low air loss and air-fluidized beds have shown effectiveness in both prevention and healing, cost, availability, and potential adverse effects warrant careful consideration [114]. This emphasizes that future trials should incorporate time-to-event outcomes, cost-effectiveness analysis, and adverse event reporting to better guide clinical decision-making. Taken together, while smart and high-tech surfaces offer exciting innovations, their optimal application should be guided by setting, patient risk level, and evolving evidence regarding comparative effectiveness.

### 7.3. Artificial Intelligence-Based Risk Prediction

Artificial intelligence (AI) algorithms have been applied to electronic health records (EHRs) and bedside monitoring data to generate real-time, patient-specific risk profiles. These tools move beyond static scales like the Braden Score by incorporating dynamic variables such as vital signs, lab values, nursing notes, and mobility status. Machine learning models have shown higher sensitivity and specificity in predicting pressure injury development compared to traditional methods, enabling earlier intervention and improved resource allocation [116]. Some systems are even embedded into hospital EHR platforms to trigger automated care pathways or clinical alerts. In rehabilitation settings, this can facilitate team-based responses—including early therapy consults, mattress upgrades, and nutritional interventions—before tissue breakdown occurs.

### 7.4. Biofeedback Tools in Therapy

Biofeedback systems have gained traction in physical and occupational therapy as tools to help patients with neuromuscular deficits recognize and modify pressure-related behaviors. Biofeedback devices can provide auditory or visual cues when dangerous pressure thresholds are reached, teaching users to reposition more effectively and consistently [117,118]. These tools are increasingly used in conjunction with powered mobility devices, integrating pressure sensors into wheelchair cushions that sync with smartphone applications [119]. In outpatient settings, this technology may empower patients to take a more active role in their care and supports long-term pressure injury prevention through behavioral change.

## 8. Implementation Challenges

Despite the availability of effective prevention and management strategies for pressure injuries, their successful implementation across care settings remains complex. These challenges span staffing, care setting variability, and financial limitations, often affecting the consistency and sustainability of prevention programs.

### 8.1. Staffing Limitations and Compliance

One of the most persistent barriers to pressure injury prevention is insufficient staffing. Evidence-based strategies such as turning patients every two hours, performing frequent skin assessments, and managing moisture require significant nursing and support staff time. However, in many acute and long-term care settings, staff-to-patient ratios are inadequate, particularly during night shifts or weekends. As a result, even well-established prevention protocols may not be reliably followed. A 2019 study highlighted that nursing workload and organizational factors directly impact pressure injury incidence in hospitalized patients [120]. Additionally, staff education is crucial; inconsistent knowledge, lack of prioritization, and poor communication among team members can lead to lapses in care, even when protocols are in place.

### 8.2. Variability in Inpatient vs. Home Care Settings

Patients with limited mobility often transition between multiple care environments, including hospitals, rehabilitation centers, skilled nursing facilities, and home. The level of support available for pressure injury prevention can vary significantly across these settings. In hospitals and rehab centers, patients typically benefit from routine monitoring and specialized equipment such as pressure-relieving mattresses. However, after discharge—especially into home care or under-resourced facilities—access to such equipment may be limited, and caregivers may lack the training or capacity to continue preventive measures. Tate et al. [121] found that patients with reduced mobility were at heightened risk of ulcer development during transitions of care, particularly when activity levels dropped or support systems weakened. This inconsistency can lead to the loss of early gains made in inpatient care and underscores the need for robust discharge planning and caregiver education.

### 8.3. Cost-Benefit Analysis of Prevention Programs

Implementing comprehensive pressure injury prevention programs requires upfront investment in staff training, equipment, and care coordination. These costs can be a deterrent, particularly in institutions facing budgetary constraints. However, pressure injuries are expensive to treat, with Stage 3 or Stage 4 ulcers potentially costing tens of thousands of dollars per patient due to extended hospitalizations, wound care supplies, and complications like infections or surgeries [7,122]. Padula et al. [123] demonstrated that preventive programs, especially those incorporating frequent repositioning and high-specification mattresses, were not only clinically effective but also cost-saving in the long term. Nevertheless, the economic benefits of prevention may not be immediately apparent in fee-for-service models, where reimbursement is based on treatment rather than outcomes. This misalignment between clinical outcomes and financial incentives can hinder the adoption of proactive prevention strategies.

## 9. Future directions

As the population of individuals with limited mobility continues to grow, especially among aging adults and those with complex medical needs, the field of pressure injury prevention must evolve to meet both individualized and system-level demands. Future efforts should focus on personalization of care, more robust outcome tracking—especially in rehabilitation or transitional environments—and greater integration of skin integrity into global functional outcome metrics.

### 9.1. Personalized Pressure Injury Prevention

Current pressure injury protocols tend to follow universal guidelines, such as fixed turning schedules or standard support surfaces. However, individual risk factors—such as body habitus, comorbidities, and nutritional status—vary widely and influence a patient’s vulnerability to skin breakdown. Future prevention strategies are moving toward personalized medicine, using predictive analytics, pressure mapping, and patient-specific data to guide individualized interventions. For example, real-time pressure distribution monitoring could inform customized turning intervals, while wearable tech may alert clinicians when thresholds for ischemia are being approached. Integrating such personalized data streams into electronic health records could allow for dynamic risk scoring that evolves with the patient’s condition rather than remaining static.

### 9.2. Better Outcome Tracking in Rehabilitation Settings

Pressure injuries are not only a marker of skin integrity but also a reflection of mobility, nutrition, caregiver support, and care coordination—all of which are central to rehabilitation medicine. However, tracking outcomes related to pressure injury prevention and healing remains inconsistent. Existing documentation often focuses more on ulcer staging than on healing trajectories, quality of life, or impact on therapy participation. Future directions should prioritize the development of standardized wound care documentation tools in electronic systems that are compatible with rehabilitation outcome scales. Additionally, longitudinal monitoring that captures re-ulceration rates post-discharge could provide valuable insights into the long-term efficacy of prevention programs. These data can also be used to support risk-adjusted benchmarking between facilities, helping to identify best practices.

### 9.3. Integration into Functional Outcome Metrics

Historically, pressure injury outcomes have been isolated from broader assessments of patient function, recovery, and quality of life. Yet for patients with limited mobility, a pressure injury can significantly delay rehabilitation progress, necessitating offloading protocols, limiting participation in gait or transfer training, and affecting discharge planning [124]. Future measurement systems should integrate skin health directly into functional metrics or emerging value-based care models. For instance, pressure injury status could be incorporated as a modifier within mobility or self-care domains, providing a more holistic view of a patient’s progress. Moreover, quality reporting programs could include pressure injury outcomes as part of bundled payment initiatives or institutional performance metrics, thereby aligning clinical priorities with financial incentives and patient-centered goals.

## 10. Conclusions

Pressure injuries remain a significant yet preventable complication among patients with limited mobility. As the healthcare system continues to shift toward value-based, patient-centered care, pressure injury prevention must be reframed as an interdisciplinary priority involving nursing, physicians, nutritionists, wound care teams, and caregivers. Evidence-based interventions such as regular repositioning, pressure-relieving devices, nutritional optimization, and skin care protocols are critical, but their success hinges on consistent implementation, individualized risk assessment, and proactive care transitions. Advances in technology, including smart sensors and AI-driven predictive tools, offer promising adjuncts, while the integration of skin integrity into functional outcome metrics could more accurately reflect patient progress and institutional quality. Ongoing research and innovation will be essential in closing gaps between guidelines and practice, ensuring that pressure injury prevention remains a central pillar of comprehensive care for vulnerable populations.

## Key Points

- Pressure injuries are preventable complications that significantly impact morbidity, length of stay, and healthcare costs in patients with limited mobility.
- Frequent repositioning protocols (e.g., q2h turning) and individualized schedules remain a cornerstone of mechanical pressure relief.
- Advanced support surfaces, including high-specification mattresses and cushions, reduce sustained tissue compression and shear.
- Moisture control, barrier creams, and skin inspection are critical elements of effective skin care strategies.
- Nutritional support, especially adequate protein, plays a vital role in prevention and healing.
- Pharmacologic interventions are limited in prevention but may assist in pain management and wound care during healing.
- Education of staff, patients, and caregivers ensures consistent preventive practices across care settings and during transitions.
- Emerging technologies like smart beds, wearable sensors, and AI-based tools offer real-time monitoring and risk prediction capabilities.
- Implementation barriers include staffing constraints, variability across care environments, and limited reimbursement for preventive care.
- Rehabilitation settings require better integration of pressure injury data into functional outcomes and long-term follow-up.
- Personalized prevention strategies and dynamic risk assessment tools will drive the next wave of innovation.
- Ongoing interdisciplinary collaboration and research are essential to improve quality, reduce disparities, and optimize patient outcomes.
